# Genomic profiling, implications for genotype-based treatment of 131 patients with phenylketonuria and characterization of novel p.Pro416Leu *PAH* variant

**DOI:** 10.1038/s41598-025-04611-2

**Published:** 2025-06-05

**Authors:** K. Klaassen, B. Kecman, S. Stankovic, J. Komazec, S. Pavlovic, Maja Stojiljkovic, M. Djordjevic

**Affiliations:** 1 Institute of Molecular Genetics and Genetic Engineering, University of Belgrade, Vojvode Stepe 444a, Belgrade, 11042 Serbia; 2 Institute for Mother and Child Healthcare of Serbia “Dr Vukan Cupic”, Belgrade, Serbia; 3 Faculty of Medicine, University of Belgrade, Belgrade, Serbia

**Keywords:** Phenylketonuria, Phenylalanine, Variant, Genotype-phenotype correlation, Therapy, Clinical genetics, Genotype, Mutation, Sequencing

## Abstract

Phenylketonuria (PKU) is the most common inborn disorder of amino acid metabolism caused by biallelic pathogenic variants in phenylalanine hydroxylase (P*AH*) gene. This study comprised genomic profiling and phenotypic characterization of 131 Serbian PKU patients along with implications for BH4 therapy. By combining Sanger sequencing, MLPA and WES re-analysis of previously unsolved cases, we identified 38 different disease causing variants in the *PAH* gene and classified them using ACMG guidelines. The most frequent variant was p.Leu48Ser (30.92%), followed by p.Arg408Trp (12.21%) and p.Ile306Val (8%). We detected one novel variant, p.Pro416Leu, which was classified as pathogenic, based on computational algorithms prediction, with destabilization as the mechanism of the effect upon PAH protein. For patients’ phenotypic classification, we used pre-treatment serum Phe level and Phe tolerance, indicating that 42% of patients had classic PKU, 22% had mild PKU and 32% were classified as MHP (4% remained unclassified). Furthermore, we performed genotype-phenotype correlation study which emphasized the inconsistency of p.Leu48Ser variant. Given that not all PKU patients benefit from genotype-based therapy (Kuvan and sepiapterin), we assessed the potential responsiveness in our patients. We categorized all detected *PAH* genotypes accordingly and with 39.1% responsive and 44.5% probably responsive found that majority of patients may respond to BH4 therapy. Our study brings the updated spectrum of molecular genetic data, variant classification, characterization of novel p.Pro416Leu variant, detailed phenotypic characteristics and BH4 responsiveness for PKU patients from Serbia, therefore contributing to better understanding of molecular landscape of PKU.

**Supplementary Information:**

The online version contains supplementary material available at 10.1038/s41598-025-04611-2.

## Introduction

Phenylketonuria (PKU; MIM# 261600) is the most common inborn disorder of amino acid metabolism. PKU is caused by biallelic pathogenic variants in the phenylalanine hydroxylase (P*AH*) gene, leading to deficiency of hepatic enzyme PAH which catalyzes the conversion of phenylalanine to tyrosine. Deficient PAH enzyme results in elevated serum levels of phenylalanine (Phe). This excess Phe crosses blood brain barrier and causes severe irreversible intellectual disability unless treatment with a low-Phe diet is established upon diagnosis^1^. The great genetic heterogeneity resulting from several thousand different variants in the *PAH* gene gives rise to a wide range of residual PAH enzyme activity and corresponding blood Phe concentrations. As a consequence, PKU phenotypes range from the mild hyperphenylalaninemia (MHP) that does not need treatment, to the most severe form - classic PKU, which requires immediate treatment in order to evade intellectual disability^2,3^. The average incidence of PKU in Europe is 1/10,000 live births, while the latest estimation for Serbia is 1:15,130^4,5^. This slightly lower incidence may reflect epidemiological distinctiveness of Serbian population with Slavic origin and prominent migratory events^6,7^ while remaining within the frame of European average^8^.

Advances in sequencing technology and the growing availability of sequencing data have highlighted the critical need for standardized interpretation and reporting of genetic test results, as well as uniform classification of genetic variants^9^. The guidelines developed by the American College of Medical Genetics and Genomics (ACMG) have been instrumental in establishing precise and standardized variant classification. This is particularly impactful for genes with a high variant burden that result in a broad spectrum of phenotypes, such as the *PAH* gene. These advancements not only enhance research into genotype-phenotype inconsistencies, one of the most complex challenges in long-studied diseases such as PKU, but also support the genetic characterization of patients.

Even though numerous genotype-phenotype inconsistencies have been noted^10–12^ two *PAH* gene variants remain the main determinant of the PKU phenotype severity. Therefore, genetic characterization of PKU patients is an invaluable approach for phenotype prediction and for implementation of optimal therapy. In addition to low-Phe diet, another therapeutic approach became available - tetrahydrobiopterin (BH4) supplementation therapy, in the form of Kuvan (sapropterin dihydrochloride)^13^. Given that not all PKU patients could benefit from it, identification of *PAH* variants is useful to predict the BH4-responsiveness based on genotype.

In our previous studies^14,15^we presented the first results on molecular and phenotypic characteristics of PKU patients from Serbia and assessed the potential benefit from BH4-supplementation therapy. In this study, we present an enlarged cohort of patients with an updated variant spectrum including variants previously not found in Serbian population, as well as one novel variant. Additionally, we present revised genotype–phenotype correlation and prediction of BH4-responsiveness on an updated genotype spectrum. Moreover, all disease causing variants in the *PAH* gene found in our population were for the first time classified according to the ACMG guidelines.

## Results

### Genotyping and ACMG classification

This study comprised molecular characterization of 131 Serbian patients with phenylketonuria. We reached a detection rate of 99.24% by identifying disease causing variants in 260 out of 262 alleles. High diagnostic efficiency was achieved by combining Sanger sequencing, WES and MLPA analysis of the *PAH* gene.

**Fig. 1:**
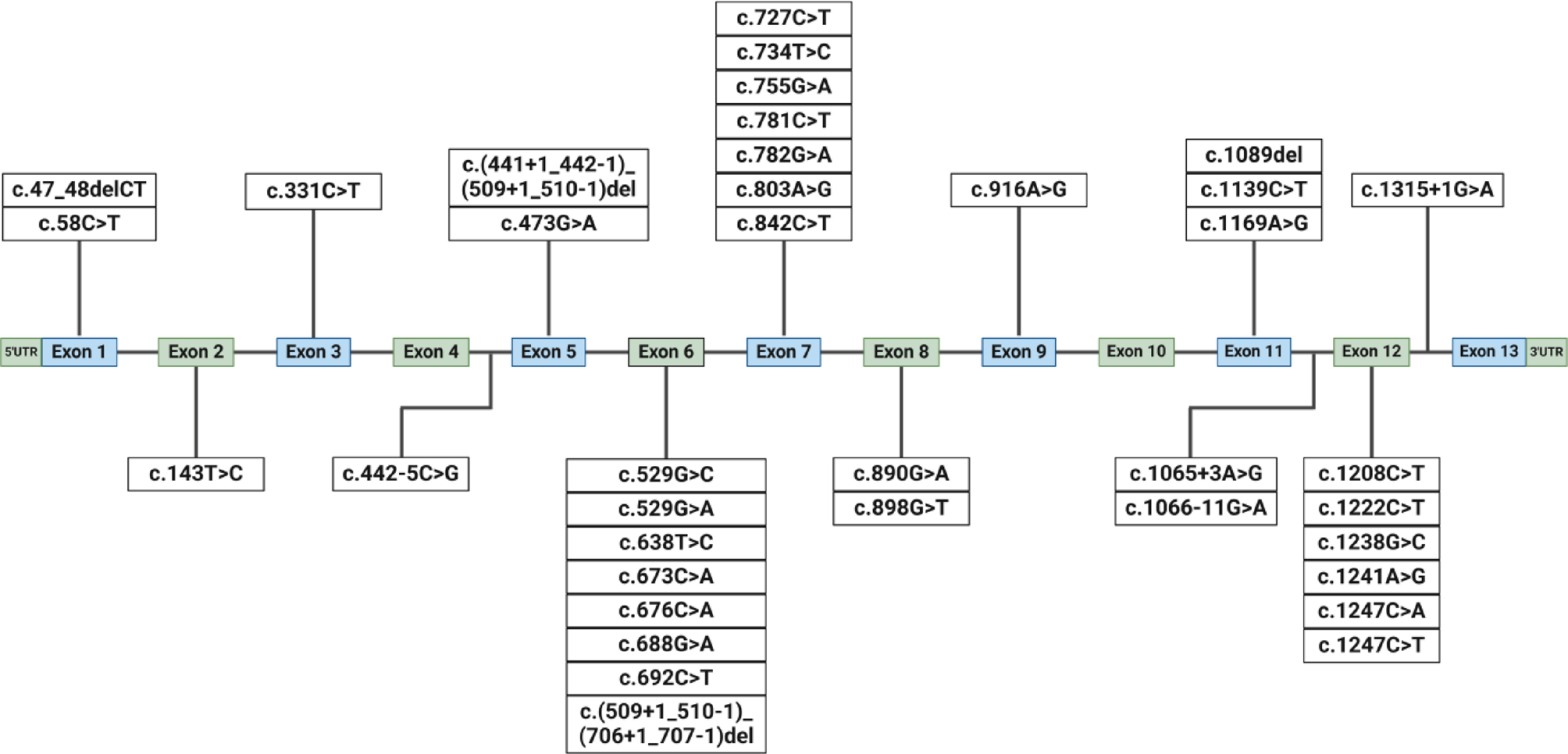
Genomic organization of the *PAH* gene and variants reported in Serbian population of patients. The 13 exons of the *PAH* gene are represented as alternating blue and green boxes, while introns are represented as black lines connecting the exons.

In this cohort of patients, we identified 38 different disease-causing variants in the *PAH* gene. Among them, 25 were missense (65.79%), 6 were splice site (15.79%), 4 were nonsense (10.5%) and 2 were frameshift indels (5.26%). The most frequent variant, c.143T > C (p.Leu48Ser), was found at 81 alleles, giving it a very high frequency of 30.92%. The second most frequent variant, c.1222 C > T (p.Arg408Trp), was present on 32 alleles (12.21%), while the third most frequent was c.916 A > G (p.Ile306Val) with a frequency of 8% and the presence on 21 alleles. Other relatively frequent variants in our cohort were c.842 C > T (p.Pro281Leu) and c.1169 A > G (p.Glu390Gly), while the remaining 33 variants were found at frequency less than 4% (Table 1). Homozygosity was observed in 28 patients (21.37%). Of them, a very high number of 20 patients were homozygous for p.Leu48Ser, followed by 2 patients homozygous for p.Arg408Thr, and each of the variants p.Ile306Val, p.Glu390Gly, p.Pro281Leu, p.Arg111Ter, p.Arg243Ter and deletion of exon 6 was found in homozygosis in single patients. The spectrum of variants across *PAH* gene exons and intronic splice regions is shown in Fig. 1.

**Table 1:** Variant spectrum with frequency and APV values found in PKU patients from serbia.

Variant	Number of alleles	Relative frequency (%)	Type of variant	APV	ACMG
c.143T > C p.Leu48Ser	81	30.92	Missense	2	P
c.1222 C > T p.Arg408Trp	32	12.21	Missense	0	P
c.916 A > G p.Ile306Val	21	8.02	Missense	9.8	P
c.842 C > T p.Pro281Leu	12	4.58	Missense	0	P
c.1169 A > G p.Glu390Gly	11	4.20	Missense	6.8	P
c.782G > A p.Arg261Gln	10	3.82	Missense	1.5	P
c.529G > C p.Val177Leu	9	3.44	Missense	7.4	P
c.473G > A p.Arg158Gln	8	3.05	Missense	0	P
c.1208 C > T p.Ala403Val	8	3.05	Missense	9.7	P
c.898G > T p.Ala300Ser	6	2.29	Missense	9.7	P
c.441 + 5G > T	5	1.91	Splice site	0	P
c.1241 A > G p.Tyr414Cys	5	1.91	Missense	5.1	P
c.673 C > A p.Pro225Thr	4	1.53	Missense	0	P
c.734T > C p.Val245Ala	4	1.53	Missense	9.9	P
c.58 C > T p.Gln20Ter	3	1.15	Nonsense	0	P
c.331 C > T p.Arg111Ter	3	1.15	Nonsense	0	P
c.442–5 C > G	3	1.15	Splice site	6.1	LP
c.676 C > A p.Gln226Lys	3	1.15	Missense	0	P
c.781 C > T p.Arg261Ter	3	1.15	Nonsense	0	P
c.1066-11G > A	3	1.15	Splice site	0	P
c.1247 C > A p.Pro416Gln	3	1.15	Missense	2.5	P
c.1315 + 1G > A	3	1.15	Splice site	0	P
c.(509 + 1_510-1)_(706 + 1_707-1)del	2	0.76	Deletion	0	P
c.692 C > T p.Ser231Phe	2	0.76	Missense	0	P
c.727 C > T p.Arg243Ter	2	0.76	Nonsense	0	P
c.1139 C > T p.Thr380Met	2	0.76	Missense	9.9	P
c.47_48delCT p.Ser16Ter	1	0.38	Frameshift	0	P
c.(441 + 1_442-1)_(509 + 1_510-1)del	1	0.38	Deletion	0	P
c.529G > A p.Val177Met	1	0.38	Missense	10	P
c.638T > C p.Leu213Pro	1	0.38	Missense	0.2	P
c.688G > A p.Val230Ile	1	0.38	Missense	9.8	P
c.755G > A p.Arg252Gln	1	0.38	Missense	0	P
c.803 A > G p.Tyr268Cys	1	0.38	Missense	0	P
c.890G > A p.Arg297His	1	0.38	Missense	10	P
c.1089del p.Lys363AsnfsTer37	1	0.38	Frameshift	0	P
c.1065 + 3 A > G	1	0.38	Splice site	0	P
c.1238G > C p.Arg413Pro	1	0.38	Missense	0	P
c.1247 C > T p.Pro416Leu	1	0.38	Missense	NA	P
Uncharacterized	2	0.76	–	–	–
Total	262	–	–	–	–

*APV -* allelic phenotype value, *ACMG -* American College of Medical Genetics and Genomics, *P -* pathogenic, *LP -* likely pathogenic, *NA -* non available, *Uncharacterized -* no disease causing variant detected on the allele.

MLPA analysis identified a homozygous deletion of exon 6 in one patient (Fig. S1A). The deletion, encompassing both MLPA probes for the exon 6, was confirmed to be inherited from unaffected parents, both carrying the detected deletion in a heterozygous state. A heterozygous deletion of exon 5 was detected in another patient (Fig. S1B), who already had one variant (p.Ile306Val) identified by sequencing (parental samples not available for segregation analysis). Both large deletions detected by MLPA were considered to be pathogenic given that the whole exon was deleted. Following MLPA analysis, two patients remained who lacked two disease-causing variants on both alleles of the *PAH* gene. In order to exclude possible BH4 deficiency (with a concurrent heterozygosity in the *PAH* gene), we performed WES for these patients. WES analysis has shown that neither of these patients had pathogenic, likely pathogenic, or variants of uncertain significance (VUS) above 50% posterior probability in genes associated with hyperphenylalaninemia (*GCH1*, *PCBD1*, *PTS*, *QDPR*, *SPR* and *DNAJC12*).

All detected variants were classified using ACMG guidelines (Table 1). Out of 36 variants detected by Sanger sequencing, 35 were classified as pathogenic, and only one variant, c.442–5 C > G, was classified as likely pathogenic. Variant c.442–5 C > G was detected in three patients, including a pair of siblings. Furthermore, for all variants detected in this study, we added APV (allelic phenotype value) from the BIOPKU database (Table 1) and calculated corresponding genotypic phenotype value (GPV). APV was used to define the severity of an individual variant, while for GPV, the effect of the milder variant overpowered the more severe one, thus predicting milder phenotypes for higher GPV values. For 21 (55%) variants, APV was 0. Given that large deletions generally abolish the function of the protein, both large deletions detected in this study were classified as pathogenic, and they were assigned APV 0.

**Fig. 2:**
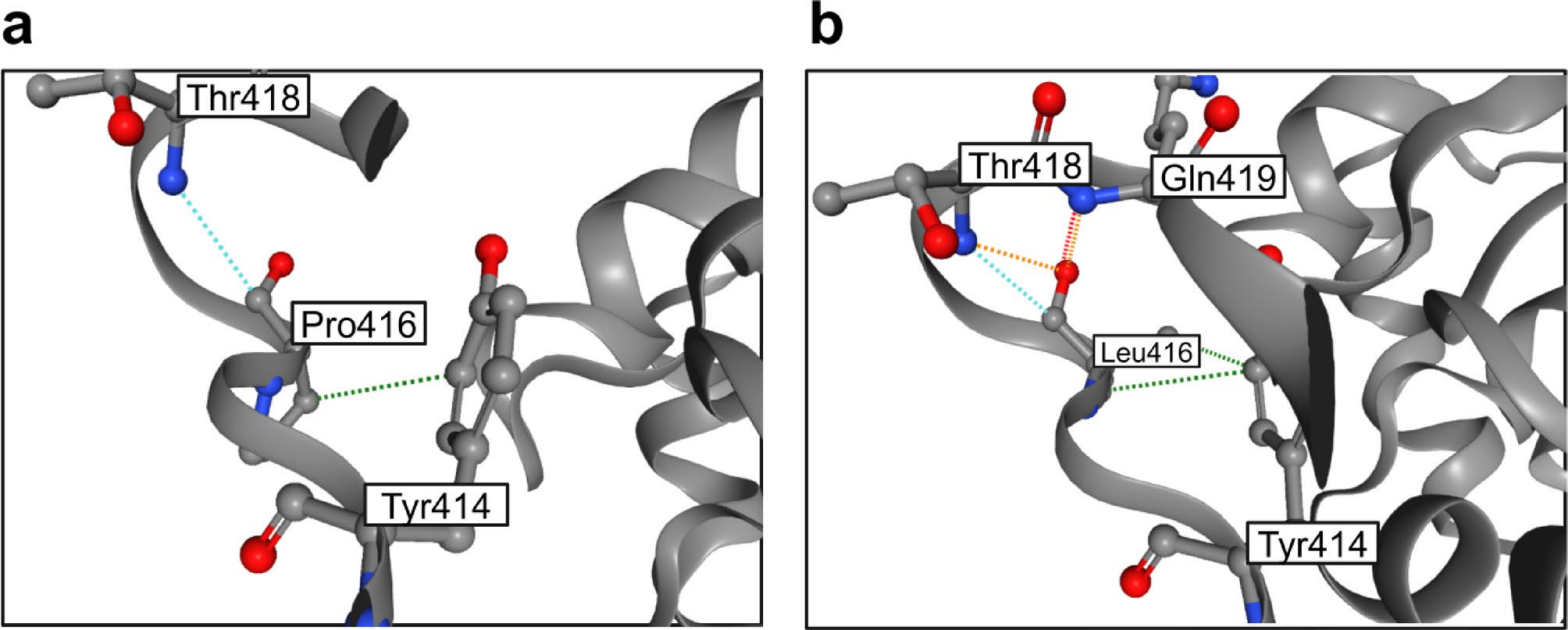
Three-dimensional molecular model of the PAH protein. Close-up view of the region harboring p.Pro416 (**a**) and p.Leu416 (**b**) was analyzed using Dynamut2. Hydrophobic interactions are shown in green dotted lines, Van der Waals interactions are shown in blue dotted lines, polar interactions are shown in orange dotted lines and hydrogen bonds are shown in red dotted lines. In case of p.Leu416, novel interactions are formed: polar interactions with Gln419 and Thr418, additional hydrophobic interaction with Tyr414 and a hydrogen bond with Gln419.

Out of 38 detected variants, there were 12 not reported in our previous studies on Serbian population—two large deletions and also 9 small changes in the *PAH* gene that have been previously described in the literature and BIOPKU database: one frameshift c.1089del (p.Lys363AsnfsTer37), two splice variants (c.441 + 5G > T and c.442–5 C > G), and six missense variants (p.Ala300Ser, p.Val245Ala, p.Gln226Lys, p.Thr380Met, p.Val230Ile and p.Tyr268Cys). Furthermore, we detected one novel variant that has not been previously described in the literature and BIOPKU database: c.1247 C > T (p.Pro416Leu), (electropherogram shown in Fig. S2). This variant was classified as pathogenic by ACMG using following criteria: PM1_Strong (located in a mutational hot spot and/or critical and well-established functional domain), PP3_Strong (multiple lines of computational evidence support a deleterious effect on the gene or gene product), PM5_Moderate (novel missense change at an amino acid residue where a different missense change determined to be pathogenic has been seen before, p.Pro416Gln) and PM2_Supporting (absent from controls in various population databases). All individual computational algorithms predicted the variant to be pathogenic, while MutPred2 additionally proposed that the mechanism of the effect upon PAH protein is destabilization. This was further confirmed by Dynamut2 and represented in Fig. 2 as a close-up view of the PAH protein region with p.Pro416 and p.Leu416 residues. Given that PKU patient harboring this change was also confirmed to have another well-established pathogenic *PAH* variant, p.Pro281Leu, the novel variant c.1247 C > T (p.Pro416Leu) was concluded to be a disease causing variant, and subsequently submitted to ClinVar (Variation ID: 3777762; Accession: VCV003777762.1).

### Phenotypic characterization and genotype-phenotype correlation study

In this study, a total of 131 PKU patients from Serbia were included, comprising 65 females and 66 males. Among them, there were four pairs of siblings. Also, four previously undiagnosed mothers of PKU patients were confirmed to have biallelic pathogenic variants in the *PAH* gene.

**Fig. 3:**
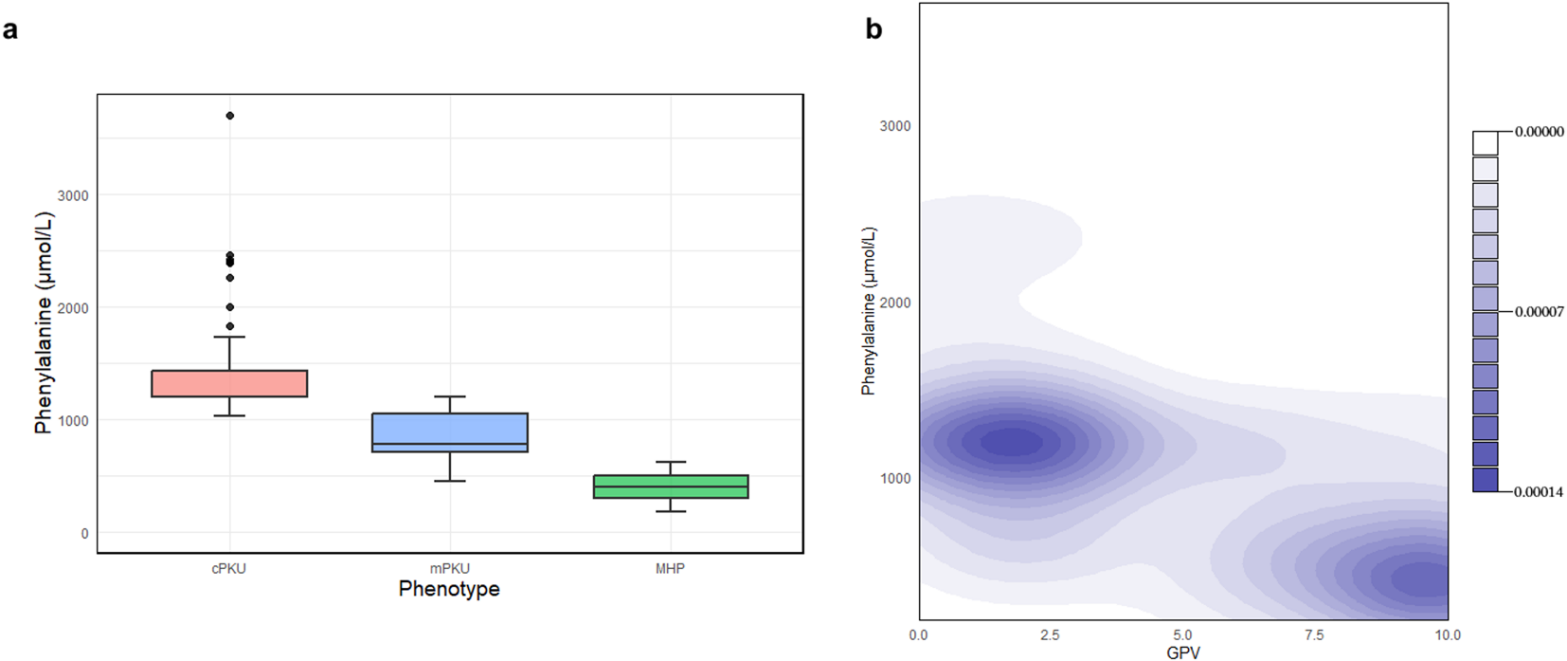
Relationship between blood Phenylalanine concentration, phenotype, and GPV. **a** Boxplot (median, 25th–75th percentile, 1.5 IQR) of the maximal blood Phe concentrations for three PKU phenotypes (cPKU, mPKU, MHP) in 125 of 131 Serbian PKU patients (for whom data was available). The black dots in the cPKU bar represent higher blood Phe concentration values, as previously documented in cPKU patients. **b** Contour plot showing the two-dimensional densities of maximal blood Phe concentrations and their associated GPVs for 125 PKU patients.

According to individual pre-treatment serum Phe level, the patients were assigned to one of the phenotypic categories: 55 (42%) had classic PKU, 29 (22%) had mild PKU and 42 (32%) were classified as MHP, while 5 remained unclassified (no data on maximal Phe level were available) (Fig. 3). Alternatively, according to individual Phe tolerance, the patients were assigned to the phenotypic categories as follows: 46 (35%) had classic PKU, 25 (19%) mild PKU and 46 (35%) MHP. Fourteen patients, for whom no data on Phe tolerance was available and were later lost to follow-up, remained unclassified.

For 96 patients, the diagnosis was obtained by neonatal screening and 22 had delayed diagnosis (after 6 months of age or later), while for 13 patients data on time of diagnosis was unavailable. All patients requiring therapy are treated with low Phe diet. Detailed phenotypic data on all patients including diagnosis age, treatment requirements, adherence to diet, and genotype is given in Table S1.

Genotype-phenotype correlation study was performed for patients homozygous for the variant and for patients compound heterozygous for a variant with an attributed APV score of 0 (presumed to be null variants and therefore associated with cPKU). The most prevalent variants, p.Leu48Ser, p.Ile306Val and p.Glu390Gly, were found to have an inconsistent effect, which was most emphasized in case of p.Leu48Ser. Variant p.Leu48Ser was shown to have an inconsistent phenotypic outcome in our previous study^15^ which was further corroborated in this enlarged cohort of patients. Patients homozygous for p.Leu48Ser were attributed to all three phenotypic categories, with three patients classified as cPKU, seven as mPKU and six as MHP. On the contrary, functionally hemizygous patients were predominantly classified as cPKU, as it was the case with 17 patients, while only three and two patients were classified as mPKU and MHP, respectively. Variant p.Ile306Val was mainly associated with MHP (eight patients), while three patients were classified as mPKU and one patient was classified as cPKU. Patients harboring p.Glu390Gly variant were also assigned to all three phenotypic categories, with two patients classified as cPKU, four as mPKU and three as MHP. Among less frequent variants, p.Arg261Gln and p.Tyr414Cys were mostly associated with cPKU, while variants p.Val177Leu and p.Pro416Gln were mostly associated with mPKU. Milder variants such as p.Ala403Val, p.Ala300Ser, p.Val230Ile and p.Thr380Met were associated with MHP in all functionally hemizygous patients from this study.

### BH4 responsiveness

**Fig. 4:**
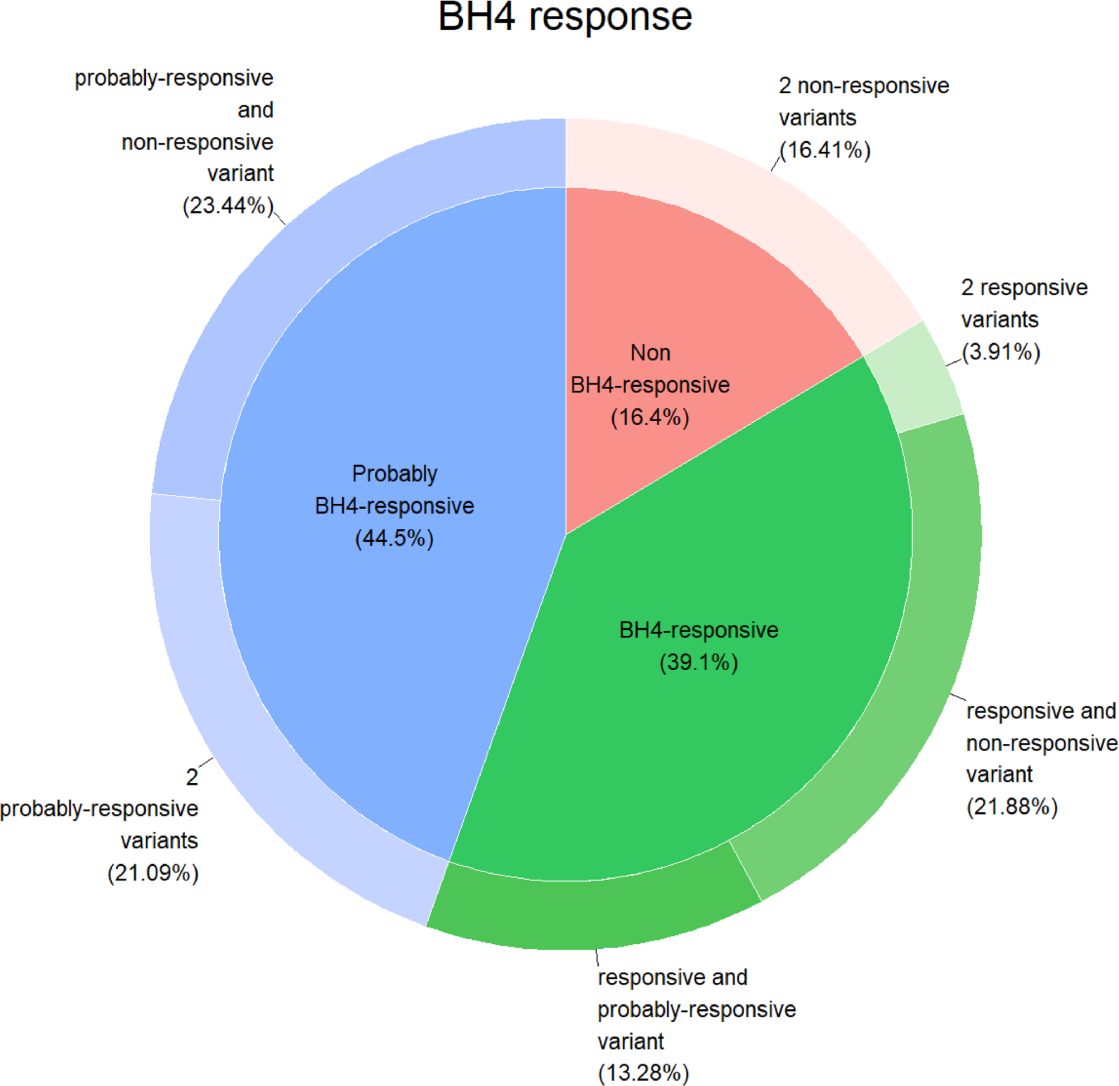
Schematic representation of BH4 response prediction based on the genotype of Serbian PKU patients.

We assessed BH4 responsiveness for genotypes found in PKU patients from Serbia. Several variants indicated as BH4 responsive that were not present in the previous study^15^ on Serbian population were detected in this enlarged cohort of patients, including p.Val230Ile, p.Val245Ala, p.Ala300Ser and p.Thr380Met, while previously reported p.Arg158Gln, p.Arg261Gln, p.Ile306Val, p.Glu390Gly, p.Ala403Val, p.Arg413Pro and p.Tyr414Cys were found with an increased occurrence^8,16–18^. Given that some variants are consistently BH4-responsive while others are observed to be inconsistently responsive, we classified the genotypes accordingly, with a distinction between genotypes with one and two BH4-responsive variants. We categorized 70 different *PAH* genotypes present in Serbia into BH4 responsive (32), probably BH4 responsive (18), and non-BH4 responsive (20), which corresponded to 39.1% of BH4 responsive patients, 44.5% probably BH4 responsive and 16.4% of non-BH4 responsive patients (Fig. 4), while detailed BH4 responsiveness based on genotype is shown in Table S2. When compared to our previous study, we observed the shift: the percentage of non-BH4 responsive genotypes has decreased from then 22.8%, which in turn led to the increase of BH4 responsive genotypes^15^. This study showed that in fact as many as 83.6% patients may respond to BH4 therapy.

## Discussion

In this study, we presented genotypic and phenotypic characterization of 131 PKU patients from Serbia. This study depicts a greatly enlarged cohort in comparison to our previous studies and it brings an updated spectrum of variants with the ACMG classification, as well as the genotype-phenotype correlation and the estimation of BH4 responsiveness.

Consistent with our previous studies^14,15^p.Leu48Ser has remained as by far the most prevalent variant in Serbia. This very high frequency of 30.92% is the highest documented in any population to date. This variant has been found sporadically in various European populations^8^but it only reached higher frequencies in populations neighboring Serbia such as in Croatian with 14.3%^8^, Romanian with 9.3%^19^ but surprisingly only 2% in Slovenia^20^. Given its unexpectedly high frequency in Serbia, it may perhaps be presumed that it arouse in this particular population, but a haplotype study indicated that it was actually introduced to Serbia from populations with diverse genetic backgrounds^7^. The most common *PAH* variant across the globe, p.Arg408Trp, came as the second most frequent for Serbian population. With around 23% as an overall frequency^2,8^but substantially higher in Slavic populations, it reaches its peak at the north Baltic region, with 82.6% for Estonia^8^ and 70% for Latvia^21^. As for neighboring Slavic populations, Croatia has a high frequency of 31.4%, but Slovenia has almost identical to Serbia − 12.5%^8^. Variant p.Ile306Val took the third place at PKU alleles from our patients’ cohort. Bearing in mind its rather low worldwide frequency of just 0.5%^8^, it would be interesting to carry out haplotype analysis to determine whether it had similar fate to p.Leu48Ser and if so, from which populations it had been imported. The remaining less frequent variants account for not more than 5% of all alleles, with twelve variants found only at individual chromosomes (so called “private” variants). The infrequent variants for Serbian population include the ones that are nevertheless rare, but there are also ones that reach somewhat higher frequencies in other populations, such as p.Thr380Met^22,23^. Interestingly, the second most prevalent variant worldwide reported by Hillert et al. in 2020, c.1066-11G > A, designated as the “Mediterranean mutation”, was found on only three chromosomes in our study, whereas p.Arg261Gln, which came third, has comparable but slightly lower frequency in Serbia. Among all variants detected in this study, around one third were not reported in our previous studies^14,15^which illustrates the ever growing variety of the *PAH* locus. Taken altogether, with 38 variants combined into 70 different genotypes for 131 patients and the relatively low homozygosity rate, a rather high degree of genetic diversity within the Serbian population can be noted, with the surprising predominance of p.Leu48Ser.

In two unrelated patients, we detected large deletions. In a late diagnosed patient with profound intellectual disability, the routine amplification of *PAH* gene exons prior to Sanger sequencing repeatedly failed to amplify exon 6, prompting a possible homozygous deletion of the entire exon. MLPA analysis confirmed the deletion of the exon 6 in the homozygous state, with both parents being heterozygous for the deletion. Another patient, with only one disease causing variant detected (p.Ile306Val), was shown to have a heterozygous deletion of the exon 5. Single-exon deletions encompassing exon 5 of the *PAH* gene were previously described in the literature and were indicated to have founder effects of Slavic (Czech) origin^24, ^also they were recurrently found in other Slavic populations^20,25^. On the other hand, a deletion of exon 6 was reported in the neighboring Romanian population^19^. These large deletions lead to loss of the *PAH* coding sequence segments, introducing frameshift, and therefore represent null variants. Large deletions in the *PAH* gene are considered to be rare, comprising only around 1–2% of all disease causing variants^20, ^even though the overall frequency of large deletions in some populations could be as high as 9%, as in Korean PKU patients with a recurrent deletion^26^. However, in order to achieve comprehensive molecular characterization and high variant detection rate, we highlighted the importance of MLPA analysis for cohort of PKU patients in this study.

In one of our patients, we detected a novel variant that has not been previously described in the literature nor in the BIOPKU database: c.1247 C > T (p.Pro416Leu). This variant was classified as pathogenic by ACMG through several criteria where multiple lines of evidence supported its deleterious effect. Individual prediction algorithms agreed on the pathogenicity of the variant, as MutPred2 additionally provided the putative mechanisms by which this change impairs PAH protein function, where the ones with highest probability included altered ordered interface, gain of strand and altered coiled coil. Dynamut2 also predicted destabilizing interactions upon the change to leucine at 416 position of the PAH protein. Given that the vast majority of the missense variants impair proper folding therefore leading to the destabilization of the PAH protein, and that PKU is essentially considered a “conformational” disease^3,27, ^it is highly likely that p.Pro416Leu can be considered a “misfolding” variant. Moreover, the presence of another pathogenic variant (p.Pro281Leu) in the patient further supports the pathogenicity of c.1247 C > T (p.Pro416Leu), making it very probable that it is in fact disease causing.

Interestingly, in one of two patients with only one detected pathogenic variant in the *PAH* gene (p.Leu48Ser), we detected a c.−30 A > T variant in the 5′ untranslated region of the *PAH* gene. This variant was previously described in a patient in whom only one pathogenic variant in the *PAH* gene was found, so it was hypothesized whether this change represents a disease causing variant or is it just a rare benign variant^28^. The variant was not found in population databases nor was it found in 100 Serbian control samples. Segregation analysis for our patient showed that c.−30 A > T was inherited maternally while the other *PAH* variant, p.Leu48Ser, was inherited paternally. Given that this patient did not have any other disease causing variants in any of the hyperphenylalaninemia genes, this rare promoter variant should be further studied. Firstly, the presence of any other deep intronic variants in the *PAH* gene that could be disease causing should be excluded. Subsequently, in order to clarify its potential effect on *PAH* gene function, functional promoter activity assays and transcriptional analyses would be crucial for this variant.

In this study, we did not find any disease causing variants in the genes associated with hyperphenylalaninemia (*GCH1*, *PCBD1*, *PTS*, *QDPR*, *SPR* and *DNAJC12*). HPA caused by BH4 deficiency is a result of biallelic pathogenic variants in genes encoding enzymes required for biosynthesis or regeneration of tetrahydrobiopterin and corresponds to around 1–2% of all HPA cases^29^. In one of our previous studies, a patient from Serbia presenting with HPA was confirmed to have a PTS deficiency^30^ and to date remains the only patient with any BH4 deficiency. Additionally, a recently discovered HPA due to the deficiency of DNAJC12, a heat shock co-chaperone family member interacting with aromatic amino acids hydroxylases^31^ further underlies the need for genetic reassessment of the patients with elevated phenylalanine but without biallelic pathogenic variants in the *PAH* gene. For our two patients with only one pathogenic *PAH* variant, we performed WES and thus excluded both BH4 or DNAJC12 deficiency.

With the ever increasing outputs of high-throughput sequencing technologies, the number of genetic variants detected has skyrocketed, putting their precise interpretation an imperative need in personalized medicine, clinical decisions, genetic counseling, and novel individualized therapy approaches. The American College of Medical Genetics and Genomics has developed the ACMG guidelines in 2015, with the intention of standardizing the assessment of the clinical significance of genetic variants^9^. This classification method defines a framework for classification, where variants fall under following categories: pathogenic, likely pathogenic, uncertain significance, likely benign and benign, guided by a number of criteria with levels of evidence described as very strong, strong, moderate and supporting. For rare Mendelian disorders like PKU, the ACMG guidelines have become a cornerstone in interpreting how genetic alterations contribute to the disease, while more recent updates from the Association for Clinical Genomic Science have further improved the classification^32^. To date, more than 3371 different variants with diverse levels of pathogenicity in the *PAH* gene have been detected (http://www.biopku.org, accessed August 9th, 2024). Given that variants of unknown significance often present major challenges for genetic evaluation, a recent study reported successful reclassification of great number of VUS variants in the *PAH* gene, thus significantly improving future assessment of PKU patients^3^.

All variants detected in our study were classified using ACMG guidelines, where all but one were classified as pathogenic. Similar to other genes associated with metabolic diseases, null (nonsense, frameshift and splice site) variants in the *PAH* gene carry high scores in ACMG classification and therefore are all classified as pathogenic. Notably, most of the uncharacterized missense variants in the *PAH* gene would also be classified as pathogenic, most of them fulfilling criteria for location in critical protein regions, absence from population databases or computational prediction. Additionally, given the high number of already described pathogenic variants, many novel variants affect known residues, giving them additional pathogenicity points. The novel variant reported in this study, p.Pro416Leu, scored 11 points and was therefore classified as pathogenic even though it has never been described in the literature. On the other hand, splice region variants score much less, even in case of already known variants established as disease causing, as it was the case with c.442–5 C > G. This variant was first classified as VUS even though ClinVar classified it as Pathogenic/Likely Pathogenic but later classified as Likely Pathogenic by adjusting the criteria. Given that other similar variants might also be disease causing but improperly classified, one should bear in mind that each variant classification needs to be fine-tuned to comprehensively characterize all *PAH* pathogenic variants.

In almost one century long history of phenylketonuria, classic PKU still stands as the hallmark of the phenotype. Out of three phenotypic categories, cPKU was found to be the most frequent in most populations studied, with as high as 81% of all patients classified as cPKU in eastern Europe, but nevertheless, Serbia was among the few countries with rates of < 50% for cPKU^8^. In our study, use of two different parameters (pre-treatment serum Phe level and Phe tolerance), corroborated that finding and indeed only up to 42% of our patients had classic PKU. Taking into account the discrepancy between these two classification methods, it was shown that for some patients Phe tolerance better depicts the phenotype, being a more realistic predictor of patients’ ability to metabolize Phe from the food^15^. Taken together with the inconsistency of guidelines which parameter should be used for classification of PKU patients, it is useful to combine these two methods.

With the introduction and implementation of newborn screening programs, the world has seen a shift towards milder forms of PKU, with the concomitant rise in frequency of milder *PAH* variants^33^. The similar could be seen for our cohort of patients, where late diagnosed (and therefore unfortunately late treated) patients tend to have more severe phenotype with profound intellectual disability, whereas for younger patients diagnosed by newborn screening we could observe the increase in MHP phenotype, particularly the ones with no dietary requirements needed. Accordingly, in this updated variant spectrum, we noted surge in frequency of milder variants such as p.Ile306Val, p.Glu390Gly, p.Ala403Val and p.Ala300Ser. It is evident that the improvements in the implementation of newborn screening will unveil more mild *PAH* variants, further expanding the diversity of the *PAH* locus.

Our previous study has pinpointed to the genotype-phenotype inconsistencies for p.Leu48Ser variant^15^. Given that the enlarged cohort from this study included more patients with p.Leu48Ser variant (both homozygous and functionally hemizygous), these inconsistencies are further emphasized. In the present study, it was shown that functionally hemizygous patients had more severe phenotypes, with the vast majority classified as cPKU, whereas for p.Leu48Ser homozygotes we noted an increase in milder phenotypes. Patients homozygous for p.Leu48Ser were found in all three phenotypic categories, ranging from typical cPKU with strict dietary regimen to outliers such as the ones who were late diagnosed and untreated but surprisingly evaded intellectual disability. These patients represent an ideal group for studying not just genotype-phenotype inconsistencies, but also additional factors that contribute to final PKU phenotype, such as novel modifier genes. Recently, a modifier gene family associated with synaptic transmission has been proposed in our study on untreated PKU patients without intellectual disability^12^. Discovery of novel modifier genes may shed new light on pathophysiology of PKU and could lead to better understanding of the brain dysfunction mediated by high Phe levels.

The observed rise in milder *PAH* variants in our cohort has led to a shift in the BH4 responsiveness. This study revealed that as many as 83.6% (with 39.1% responsive and 44.5% probably responsive) patients may benefit from BH4 therapy. This percentage is higher than the average for Europe^34^and may be in part explained by population-specific factors. The high overall frequency of variants with acknowledged substantial residual PAH activity such as p.Ile306Val, p.Glu390Gly, p.Arg261Gln, p.Arg158Gln, p.Ala403Val and p.Ala300Ser^3^ along with exceptionally high p.Leu48Ser occurrence (the highest ever reported in European populations^8^) has expanded the predominance of BH4 responsive variants and genotypes. Nevertheless, BH4 loading test remains the only way to precisely determine BH4 responsiveness for individual patients. In case of unavailability of BH4 loading test, *in vitro* assays can provide preliminary results for single variants. Therefore, in our previous studies, we tested BH4 responsiveness alongside functional characterization of novel *PAH* variants^35,36^. BH4 supplementation therapy, in the form of sapropterin dihydrochloride and brand name Kuvan has been in use since its FDA approval in 2007. However, recent studies show that sepiapterin, as the natural precursor of BH4, has more advantages over BH4 in terms of stability and transport efficacy^37^. Sepiapterin was also shown to be effective in the reduction of blood Phe with a fast onset^38^. Therefore, the research aimed at the development of enhanced therapeutic agents for PKU continues.

In the era of precision medicine, genetic analysis plays a crucial role in the understanding and management of rare metabolic diseases. For PKU, a hallmark of a rare metabolic disease, it provides definite confirmation of the diagnosis and distinguishes different forms of hyperphenylalaninemia. By identifying specific underlying genetic variants, as well as modifier genes that influence the phenotype, healthcare providers can offer more accurate diagnoses and tailored treatments, such as BH4 and sepiapterin. Understanding these genetic interactions not only enhances the efficacy of therapeutic interventions but also opens avenues for the development of novel targeted therapies, transforming the landscape of rare metabolic disease treatment and offering hope for more personalized and effective care. Furthermore, our study highlights the significance of periodic re-analysis of previously unsolved rare disease cases using the latest methodologies, in order to end their diagnostic odyssey and expand treatment opportunities.

## Methods

In this study, a total of 131 PKU patients from Serbia were included. The cohort of 61 patients had been previously described^14,15^. Since 2013, additional 70 PKU patients were diagnosed and included in this study. Patients were identified through the national newborn screening program (established in Serbia in 1982) or during genetic counseling at the Mother and Child Health Care Institute of Serbia “Dr Vukan Cupic” in Belgrade. Furthermore, four maternal samples were investigated as a way to ascertain possible diagnosis of PKU.

This study has been approved by the Ethics Committee of the Mother and Child Health Care Institute of Serbia “Dr Vukan Cupic” in Belgrade, Serbia (Number 31/2020 dated September 25th, 2020.) and Research Ethics Committee of the Institute of Molecular Genetics and Genetic Engineering, University of Belgrade (O-EO-017/1/2020 dated 03.09.2020.) and has therefore been performed in accordance with the ethical standards laid down in the 1964 Declaration of Helsinki and its later amendments. Informed consent was obtained from all individual participants included in the study.

Patients were classified into three phenotypic categories in accordance with two different parameters, pre-treatment plasma Phe level and Phe tolerance: classic PKU (pretreatment Phe > 1200 µmol/L; Phe tolerance—250–350 mg/day), mild PKU (pretreatment Phe 600–1200 µmol/L; Phe tolerance—350–600 mg/day), and MHP (pretreatment Phe < 600 µmol/L; Phe tolerance—> 600 mg/day)^39^. Phe tolerance was evaluated during hospitalization or as agreed with the patient/parents. Maximal Phe level represents the highest pretreatment blood Phe concentration (µmol/L). All patients were further referred to the Institute of Molecular Genetics and Genetic Engineering, University of Belgrade, where molecular genetic analysis was performed.

Genomic DNA was isolated from peripheral blood using QIAamp DNA-Blood-Mini-Kit (QIAGEN, Hilden, Germany). All 13 exons with their flanking intron regions of the *PAH* gene (reference transcript NM_000277.3), as well as the promoter region, were amplified and sequenced using BigDye Terminator v3.1 Cycle Sequencing Kit on the Applied Biosystems SeqStudio Genetic Analyzer (Thermo Fisher Scientific, Waltham, Massachusetts, USA). Primers used for amplification and sequencing reactions are presented in Table S3. Patient samples with no/only one disease causing variant in the *PAH* gene detected were further analyzed using MLPA (Multiplex Ligation-dependent Probe Amplification). MLPA was performed according to the manufacturers’ protocol using SALSA MLPA probemix kit P055-D1 (MRC Holland, Amsterdam, The Netherlands). The probemix P055-D1 includes 38 MLPA probes, of which 22 probes cover the *PAH* gene. Samples of suspected patients and three reference controls were used in each run with 100 ng DNA. The final PCR products were run on SeqStudio Genetic analyzer (Thermo Fisher Scientific, Waltham, Massachusetts, USA) and obtained data was analyzed by using Coffalyser.Net software (MRC Holland, Amsterdam, The Netherlands). Threshold ratios for detecting deletions and or duplications were applied according to the manufacturer’s recommendations. The reference range for normal copy number was 0.8–1.2, therefore ratio of 0.40–0.65 indicated a heterozygous deletion, while ratio of 0.0 indicated a homozygous deletion. Unsolved cases were defined as patients in whom only one or no pathogenic variant could be identified in the *PAH* gene following Sanger sequencing and MLPA analysis. These two samples were subjected to Whole Exome Sequencing (WES) for further genetic evaluation. Library preparation was performed using 50 ng of genomic DNA according to Illumina DNA Prep with Exome 2.0 Plus Enrichment kit protocol. Sequencing (with 150-bp paired-end runs) was performed on Illumina NextSeq 2000 System (Illumina, San Diego, CA, USA), with alignment and analysis performed against the GRCh38/hg38 reference genome assembly. Variants passing quality call (QC) filters (quality score > Q20, read depth > 20, percentage of variant frequency for the minor allele > 20%) and with minor allele frequencies < 1% in The Genome Aggregation Database (gnomAD) were further analyzed. Systematic interpretation of variants was performed using VarSome Clinical (Saphetor, Lausanne, Switzerland). All variants detected were classified using ACMG guidelines^9^whereas novel variant was additionally characterized using various in silico prediction algorithms, including MutPred2^40^ and MutationTaster^41^. For structure modeling of the novel variant, we used the crystal structure of human PAH (PDB: 2PAH)^42^. Dynamut2 was used to predict the impact of the variants on protein stability^43^. For data processing tidyr (version 1.3.1)^44^ and dplyr (version 1.1.4)^45^ R packages were used, while figures were created using ggplot2 (version 3.5.1)^46^ R package.

## Electronic supplementary material

Below is the link to the electronic supplementary material.

Supplementary Material 1

## Data Availability

New variant is submitted to ClinVar with the submission number SUB15184086, Variation ID: 3777762; Accession: VCV003777762.1. Genetic and phenotype data of the entire cohort of patients are provided within the manuscript or supplementary information files.
