# Supplementary material for: Genomic profiling, implications for genotype-based treatment of 131 patients with phenylketonuria and characterization of novel p.Pro416Leu PAH variant
Source: Sci Rep. 2025 Jun 5;15:19745. doi: 10.1038/s41598-025-04611-2 (PMC12141581; doi:10.1038/s41598-025-04611-2)
Supplement: Supplementary file 1 — Supplementary Material 1 [file 41598_2025_4611_MOESM1_ESM.pdf]

SUPPLEMENTARY MATERIALS

Genomic profiling, implications for genotype-based treatment of 131 patients with phenylketonuria and characterization of novel p.Pro416Leu *PAH* variant

Klaassen K[1], Kecman B[2], Stankovic S[1], Komazec J[1], Pavlovic S[1], Stojiljkovic M[1]*, Djordjevic M[2,3]

[1]Institute of Molecular Genetics and Genetic Engineering, University of Belgrade, Belgrade, Serbia

[2]Institute for Mother and Child Healthcare of Serbia „Dr Vukan Cupic“, Belgrade, Serbia

[3]Faculty of Medicine, University of Belgrade, Belgrade, Serbia

*Corresponding author: Maja Stojiljkovic, PhD

Institute of Molecular Genetics and Genetic Engineering, University of Belgrade

Vojvode Stepe 444a, 11042 Belgrade, SERBIA

Tel: +381-64-2202-373, Fax: +381-11-3975-808

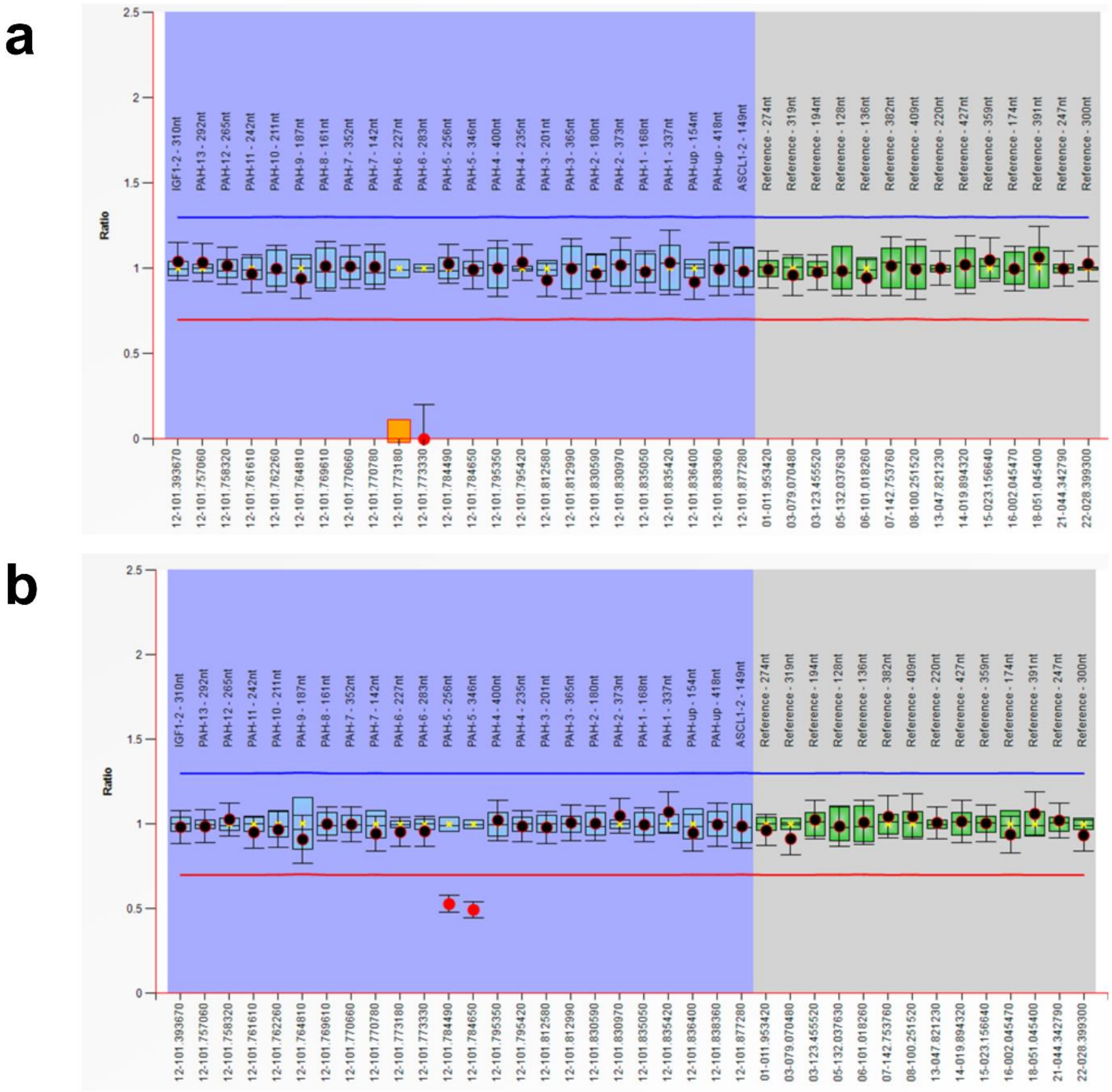

Figure S1. Ratio charts of Multiplex Ligation-Dependent Probe Amplification (MLPA) analysis results for two patients with deletions of exons 6 and 5 in the *PKU* gene, respectively. (A) a homozygous deletion of exon 6; (B) a heterozygous deletion of exon 5. The normal sample ratio range is 0.8–1.2 (between the blue and the red line); ratio between 0.0–0.4 indicates a homozygous deletion and a ratio between 0.4–0.65 indicates a heterozygous deletion.

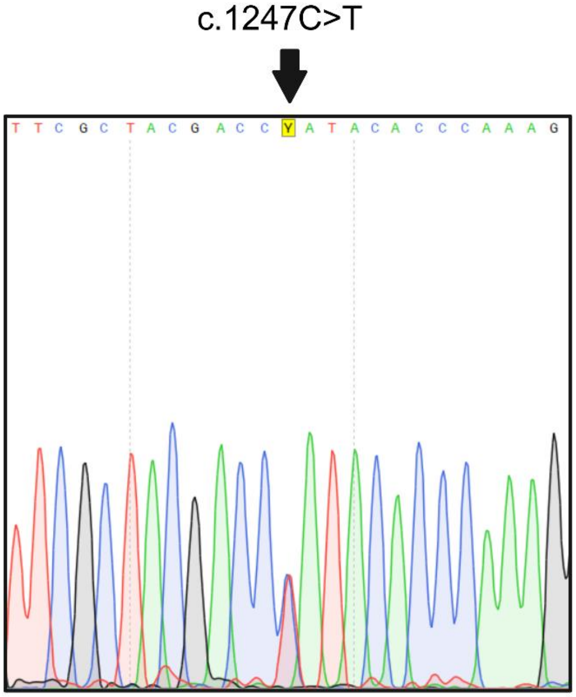

Figure S2. Electropherogram representing the novel variant c.1247C>T (p.Pro416Leu) in heterozygous state.

Table S1. Phenotypic characteristics of 131 Serbian PKU patient

Patient number	Variant 1	Variant 2	Max Phe (µmol/L)	Phe Tolerance (mg/day)	Phenotype (Max Phe)	Phenotype (Phe Tolerance)	IQ	IQ descriptive	Diagnosis	Treatment	Comments
1	c.1222C>T p.Arg408Trp	c.1065+3A>G	720	N/A	mPKU	N/A	110	HI	DBNS	LPD	On LPD from the first month to the age of 12 years
2	c.842C>T p.Pro281Leu	c.673C>A p.Pro225Thr	>1200	300	cPKU	cPKU	60	LID	LD	PCD	Late diagnosis, in the second year of life; LPD was advised but poorly controlled
3	c.1222C>T p.Arg408Trp	c.1222C>T p.Arg408Trp	>1200	300	cPKU	cPKU	95	AI	DBNS	LPD	LPD introduced in the second month
4	c.143T>C p.Leu48Ser	c.473G>A p.Arg158Gln	>1200	280	cPKU	cPKU	85	LAI	DBNS	LPD	LPD introduced in the first month, well controlled for the first 5 years
5	c.1315+1G>A	c.890G>A p.Arg297His	780	N/A	mPKU	N/A	95	AI	N/A	N/A	Not on LPD
6	c.143T>C p.Leu48Ser	c.842C>T p.Pro281Leu	>1200	400	cPKU	mPKU	59	LID	LD	LPD	LPD introduced at 6 years of age, mild intellectual disability, no epilepsy
7	c.143T>C p.Leu48Ser	c.529G>C p.Val177Leu	600	500	MHP	mPKU	95	AI	DBNS	LPD	LPD introduced from the first month
8	c.143T>C p.Leu48Ser	c.143T>C p.Leu48Ser	>1200	310	cPKU	cPKU	77.5	BIF	LD	LPD	LPD introduced at one year of age
9	c.143T>C p.Leu48Ser	c.1222C>T p.Arg408Trp	>1200	N/A	cPKU	N/A	N/A	N/A	N/A	N/A	N/A
10	c.1169A>G p.Glu390Gly	c.781C>T p.Arg261Ter	660	650	mPKU	MHP	90	AI	LD	PCD/ND	LPD introduced from 1 year until 2 years of age
11	c.1222C>T p.Arg408Trp	c.755G>A p.Arg252Gln	N/A	N/A	cPKU	N/A	N/A	N/A	N/A	N/A	N/A
12	c.782G>A p.Arg261Gln	c.47_48delCT p.Ser16Ter	>1200	300	cPKU	cPKU	92	AI	DBNS	LPD	On LPD from 1.5 months of age
13	c.1169A>G p.Glu390Gly	c.473G>A p.Arg158Gln	540	700	MHP	MHP	100	AI	DBNS	LPD/NND	On LPD until 3 months of age
14	c.1222C>T p.Arg408Trp	c.673C>A p.Pro225Thr	>1200	280	cPKU	cPKU	100	AI	DBNS	LPD	On LPD from the first month

15	c.782G>A p.Arg261Gln	c.916A>G p.Ile306Val	720	750	mPKU	MHP	110	HI	DBNS	LPD/NND	LPD for the first 5 months
16	c.143T>C p.Leu48Ser	c.782G>A p.Arg261Gln	>1200	300	cPKU	cPKU	50	MID	DBNS	PCD	LPD introduced at 4 months, poorly controlled, delayed psychomotor development, epilepsy
17	c.1222C>T p.Arg408Trp	c.1169A>G p.Glu390Gly	>1200	400	cPKU	mPKU	80	BIF	DBNS	LPD/ ND	LPD from 3 months to 3.5 years
18	c.143T>C p.Leu48Ser	c.473G>A p.Arg158Gln	>1200	300	cPKU	cPKU	90	AI	DBNS	LPD	LPD introduced in the first month
19	c.143T>C p.Leu48Ser	c.1222C>T p.Arg408Trp	>1200	300	cPKU	cPKU	84	BIF	DBNS	LPD/ PCD	LPD introduced in the first month, well controlled until about 3 years of age
20	c.842C>T p.Pro281Leu	c.1315+1G>A	2460	320	cPKU	cPKU	98	AI	DBNS	LPD	LPD introduced in the first month
21	c.143T>C p.Leu48Ser	c.842C>T p.Pro281Leu	>1500	350	cPKU	cPKU	60	LID	LD	LPD	LPD introduced late, at 20 months of age
22	c.842C>T p.Pro281Leu	c.842C>T p.Pro281Leu	>1200	340	cPKU	cPKU	30-35	SID	LD	LPD	LPD introduced from 13 months, epilepsy
23	c.331C>T p.Arg111Ter	c.331C>T p.Arg111Ter	>1200	290	cPKU	cPKU	100	AI	DBNS	LPD	LPD from the 1st month
24	c.331C>T p.Arg111Ter	c.1066-11G>A	>1200	300	cPKU	cPKU	30	SID	LD	LPD	LPD introduced at 27 months of age, epilepsy
25	c.1208C>T p.Ala403Val	/	N/A	N/A	N/A	N/A	N/A	N/A	N/A	N/A	N/A
26	c.1222C>T p.Arg408Trp	c.1169A>G p.Glu390Gly	>1200	300	cPKU	cPKU	95	AI	LD	LPD	LPD from 17 months of age
27	c.1222C>T p.Arg408Trp	c.1169A>G p.Glu390Gly	>1200	260	cPKU	cPKU	95	AI	DBNS	LPD	LPD from the 1st month of age
28	c.143T>C p.Leu48Ser	c.1222C>T p.Arg408Trp	>1200	350	cPKU	cPKU	105	HI	DBNS	LPD	LPD introduced in the first month
29	c.143T>C p.Leu48Ser	c.58C>T p.Gln20Ter	>1200	300	cPKU	cPKU	89	LAI	LD	LPD	IQ 45 before LPD, LPD introduced at 3 years of age, well controlled; after two years on LPD, IQ 74; after 5 years on LPD, IQ 89

30	c.143T>C p.Leu48Ser	c.143T>C p.Leu48Ser	1180	N/A	mPKU	N/A	N/A	N/A	N/A	N/A	N/A
31	c.529G>C p.Val177Leu	c.58C>T p.Gln20Ter	1200	600	mPKU	MHP	90	AI	DBNS	LPD	LPD introduced at 2.5 months of age
32	c.782G>A p.Arg261Gln	c.1315+1G>A	N/A	N/A	mPKU	N/A	N/A	N/A	N/A	N/A	N/A
33	c.143T>C p.Leu48Ser	c.916A>G p.Ile306Val	>1200	280	cPKU	cPKU	110	HI	DBNS	LPD	LPD introduced in the first month
34	c.781C>T p.Arg261Ter	c.916A>G p.Ile306Val	600	≥ 600	MHP	MHP	80	BIF	DBNS	LPD/ND	LPD until the end of the first year
35	c.1222C>T p.Arg408Trp	c.1169A>G p.Glu390Gly	900	400	mPKU	mPKU	75	BIF	DBNS	PCD	LPD introduced in the first month, poorly controlled
36	c.143T>C p.Leu48Ser	c.692C>T p.Ser231Phe	>1200	230	cPKU	cPKU	20	SID	LD	ND	Diagnosed at 5 years of age, not on LPD
37	c.143T>C p.Leu48Ser	c.692C>T p.Ser231Phe	>1200	240	cPKU	cPKU	25	SID	LD	ND	Diagnosed at 18 months, not on LPD
38	c.1222C>T p.Arg408Trp	c.1238G>C p.Arg413Pro	>2000	260	cPKU	cPKU	103	HI	DBNS	LPD	LPD from the first month
39	c.143T>C p.Leu48Ser	c.1222C>T p.Arg408Trp	>1200	500	cPKU	mPKU	110	HI	LD	LPD/NND	LPD from 22 months to 5 years of age
40	c.143T>C p.Leu48Ser	c.143T>C p.Leu48Ser	700	N/A	mPKU	N/A	N/A	N/A	N/A	N/A	N/A
41	c.727C>T p.Arg243Ter	c.727C>T p.Arg243Ter	1680	290	cPKU	cPKU	85	LAI	DBNS	PCD	LPD introduced in the first month, poorly controlled
42	c.143T>C p.Leu48Ser	c.916A>G p.Ile306Val	1080	210 - 610	mPKU	mPKU	35	SID	LD	ND	Developmental delay, not on LPD
43	c.143T>C p.Leu48Ser	c.1222C>T p.Arg408Trp	>1200	350	cPKU	cPKU	102.5	HI	DBNS	LPD	LPD introduced in the first month
44	c.143T>C p.Leu48Ser	c.673C>A p.Pro225Thr	2400	280	cPKU	cPKU	100	AI	DBNS	LPD	LPD introduced in the first month

45	c.143T>C p.Leu48Ser	c.143T>C p.Leu48Ser	>1200	330	cPKU	cPKU	80	BIF	DBNS	PCD	LPD introduced in the first month, poorly controlled. Mother genotype: L48S/L48S, Phe 1200; mother not on LPD; IQ 80
46	c.1222C>T p.Arg408Trp	c.529G>C p.Val177Leu	760	550	mPKU	mPKU	95-100	AI	DBNS	LPD	LPD introduced in the first month
47	c.842C>T p.Pro281Leu	c.1169A>G p.Glu390Gly	1200	400	mPKU	mPKU	95-100	AI	DBNS	LPD	LPD introduced in the first month
48	c.143T>C p.Leu48Ser	c.143T>C p.Leu48Ser	1200	400	mPKU	mPKU	110	HI	LD	LPD	LPD introduced at 11 months of age
49	c.143T>C p.Leu48Ser	c.143T>C p.Leu48Ser	1200	400	mPKU	mPKU	115	HI	LD	LPD	LPD introduced at 11 months of age
50	c.143T>C p.Leu48Ser	c.143T>C p.Leu48Ser	1200	450	mPKU	mPKU	85	LAI	DBNS	ND	Not on LPD
51	c.143T>C p.Leu48Ser	c.473G>A p.Arg158Gln	>1200	300	cPKU	cPKU	87.5	LAI	DBNS	PCD	LPD introduced in the first month, poorly controlled. Mother's Phe level 780, genotype L48S/L48S, never on LPD, IQ 80
52	c.1222C>T p.Arg408Trp	c.916A>G p.Ile306Val	1440	350	cPKU	cPKU	100	AI	DBNS	LPD	LPD introduced at 2 months of age
53	c.1222C>T p.Arg408Trp	c.1208C>T p.Ala403Val	420	≥ 600	MHP	MHP	90	AI	DBNS	NND	Not on LPD
54	c.916A>G p.Ile306Val	c.1247C>A p.Pro416Gln	780	≥ 600	mPKU	MHP	95-100	AI	DBNS	LPD/NND	LPD until 5 months of age
55	c.1066-11G>A	c.1241A>G p.Tyr414Cys	>1200	280	cPKU	cPKU	95	AI	DBNS	LPD	LPD introduced in the first month
56	c.143T>C p.Leu48Ser	c.143T>C p.Leu48Ser	900	550	mPKU	mPKU	100	AI	DBNS	LPD	LPD introduced in the first month
57	c.143T>C p.Leu48Ser	c.529G>A p.Val177Met	300	≥ 600	MHP	MHP	100	AI	DBNS	NND	Not on LPD
58	c.143T>C p.Leu48Ser	c.1222C>T p.Arg408Trp	>1200	300	cPKU	cPKU	97.5	AI	DBNS	LPD	LPD introduced in the first month
59	c.143T>C p.Leu48Ser	c.1222C>T p.Arg408Trp	>1200	340	cPKU	cPKU	100	AI	DBNS	LPD	LPD introduced in the first month

60	c.143T>C p.Leu48Ser	c.143T>C p.Leu48Ser	540	800	MHP	MHP	100	AI	DBNS	LPD	LPD introduced from the first month, well controlled
61	c.1241A>G p.Tyr414Cys	c.638T>C p.Leu213Pro	>1200	260	cPKU	cPKU	100	AI	DBNS	LPD	LPD introduced in the first month
62	c.(509+1_510-1)_(706+1_707-1)del	c.(509+1_510-1)_(706+1_707-1)del	>1200	>600	cPKU	N/A	N/A	SID	LD	ND	N/A
63	c.1222C>T p.Arg408Trp	c.1089delG p.Lys363AsnfsTer37	1211	400	cPKU	cPKU	N/A	BIF	LD	LPD	LPD introduced at 4 months
64	c.58C>T p.Gln20Ter	c.676C>A p.Gln226Lys	1200	N/A	cPKU	N/A	N/A	SID	LD	PCD/ND	Not on LPD
65	c.143T>C p.Leu48Ser	c.781C>T p.Arg261Ter	1731	300	cPKU	N/A	N/A	N/A	DBNS	LPD	LPD introduced in the first month, well controlled
66	c.1169A>G p.Glu390Gly	c.1169A>G p.Glu390Gly	569	400	MHP	N/A	N/A	N/A	DBNS	LPD	LPD introduced in the first month, well controlled
67	c.529G>C p.Val177Leu	c.1208C>T p.Ala403Val	381	N/A	MHP	N/A	95	AI	N/A	N/A	Not on LPD
68	c.676C>A p.Gln226Lys	c.916A>G p.Ile306Val	630	N/A	mPKU	N/A	100	AI	N/A	N/A	Not on LPD
69	c.143T>C p.Leu48Ser	c.734T>C p.Val245Ala	636	N/A	mPKU	N/A	95	AI	N/A	N/A	Not on LPD
70	c.143T>C p.Leu48Ser	c.143T>C p.Leu48Ser	N/A	N/A	N/A	N/A	N/A	N/A	N/A	N/A	On LPD, lost to follow up
71	c.782G>A p.Arg261Gln	c.916A>G p.Ile306Val	510	N/A	MHP	MHP	100	AI	DBNS	ND	Not on LPD
72	c.782G>A p.Arg261Gln	c.1169A>G p.Glu390Gly	N/A	N/A	N/A	N/A	N/A	AI	DBNS	ND	Not on LPD
73	c.916A>G p.Ile306Val	c.441+5G>T	450	600	mPKU	mPKU	100	AI	DBNS	LPD	LPD introduced at 11 months
74	c.143T>C p.Leu48Ser	c.143T>C p.Leu48Ser	N/A	N/A	N/A	N/A	N/A	N/A	N/A	N/A	Not on LPD
75	c.143T>C p.Leu48Ser	c.529G>C p.Val177Leu	517	1000	MHP	MHP	100	AI	DBNS	LPD	LPD introduced from the first month, well controlled

76	c.1222C>T p.Arg408Trp	c.782G>A p.Arg261Gln	1428	320	cPKU	cPKU	109	HI	DBNS	LPD	LPD introduced from the first month, well controlled
77	c.143T>C p.Leu48Ser	c.1222C>T p.Arg408Trp	2261	400	cPKU	cPKU	90	AI	DBNS	LPD	LPD introduced from the first month, well controlled
78	c.916A>G p.Ile306Val	c.1208C>T p.Ala403Val	268	N/A	MHP	MHP	100	AI	DBNS	ND	Not on LPD
79	c.143T>C p.Leu48Ser	c.442-5C>G	460	1000	MHP	MHP	114	HI	DBNS	LPD	LPD introduced from the first month
80	c.143T>C p.Leu48Ser	c.143T>C p.Leu48Ser	498	1000	MHP	MHP	93	AI	DBNS	LPD	LPD introduced from the first month, well controlled
81	c.1222C>T p.Arg408Trp	c.1222C>T p.Arg408Trp	1834	350	cPKU	cPKU	100	AI	DBNS	LPD	LPD introduced from the first month, well controlled
82	c.842C>T p.Pro281Leu	c.1247C>T p.Pro416Leu	N/A	N/A	N/A	N/A	N/A	N/A	N/A	N/A	N/A
83	c.1066-11G>A	c.1208C>T p.Ala403Val	315	N/A	MHP	MHP	100	AI	DBNS	ND	Not on LPD
84	c.143T>C p.Leu48Ser	c.898G>T p.Ala300Ser	330	N/A	MHP	MHP	100	AI	DBNS	ND	Not on LPD
85	c.734T>C p.Val245Ala	c.916A>G p.Ile306Val	188	N/A	MHP	MHP	95	AI	DBNS	ND	Not on LPD
86	c.143T>C p.Leu48Ser	c.916A>G p.Ile306Val	258	N/A	MHP	MHP	95	AI	DBNS	ND	Not on LPD
87	c.529G>C p.Val177Leu	c.1208C>T p.Ala403Val	454	800	MHP	MHP	79	BIF	DBNS	LPD	LPD introduced from the first month, well controlled, delayed psychomotor development
88	c.916A>G p.Ile306Val	c.1222C>T p.Arg408Trp	454	450	MHP	mPKU	100	AI	DBNS	LPD	LPD introduced from the first month, well controlled
89	c.143T>C p.Leu48Ser	c.1222C>T p.Arg408Trp	1425	550	cPKU	mPKU	100	AI	DBNS	LPD	LPD introduced from the first month, well controlled
90	c.143T>C p.Leu48Ser	/	969	500	mPKU	mPKU	95	AI	DBNS	LPD	LPD introduced from the first month, well controlled

91	c.143T>C p.Leu48Ser	c.782G>A p.Arg261Gln	726	500	mPKU	mPKU	100	AI	DBNS	LPD	LPD introduced from the first month, poorly controlled
92	c.143T>C p.Leu48Ser	c.734T>C p.Val245Ala	460	700	MHP	MHP	100	AI	DBNS	LPD	LPD introduced from the first month, well controlled
93	c.529G>C p.Val177Leu	c.1208C>T p.Ala403Val	303	N/A	MHP	MHP	100	AI	DBNS	ND	Not on LPD
94	c.916A>G p.Ile306Val	c.(441+1_442-1)_(509+1_510-1)del	466,1	800	MHP	MHP	105	HI	DBNS	LPD	LPD introduced at 6 months
95	c.143T>C p.Leu48Ser	c.143T>C p.Leu48Ser	623,5	1000	MHP	MHP	100	AI	DBNS	LPD	LPD introduced from the first month, well controlled
96	c.143T>C p.Leu48Ser	c.143T>C p.Leu48Ser	2421,5	500	cPKU	mPKU	100	AI	DBNS	LPD	LPD introduced from the first month, well controlled
97	c.143T>C p.Leu48Ser	c.442-5C>G	242,1	N/A	MHP	MHP	115	HI	DBNS	ND	Not on LPD
98	c.143T>C p.Leu48Ser	c.1247C>A p.Pro416Gln	1029,1	350	cPKU	cPKU	60	LID	DBNS	LPD	LPD poorly controlled, mother has PKU and was never on LPD
99	c.143T>C p.Leu48Ser	c.842C>T p.Pro281Leu	1452,9	350	cPKU	cPKU	90	AI	DBNS	LPD	LPD introduced from the first month, well controlled
100	c.916A>G p.Ile306Val	c.673C>A p.Pro225Thr	339	N/A	MHP	MHP	100	AI	DBNS	ND	Not on LPD
101	c.143T>C p.Leu48Ser	c.529G>C p.Val177Leu	787	500	mPKU	mPKU	100	AI	DBNS	LPD	LPD introduced from the first month
102	c.1241A>G p.Tyr414Cys	c.442-5C>G	242,1	N/A	MHP	MHP	100	AI	DBNS	ND	Not on LPD
103	c.842C>T p.Pro281Leu	c.441+5G>T	3692,7	350	cPKU	cPKU	95	AI	DBNS	LPD	LPD introduced from the first month, well controlled
104	c.803A>G p.Tyr268Cys	c.916A>G p.Ile306Val	490,3	650	MHP	MHP	85	LAI	DBNS	LPD	LPD introduced from the first month, well controlled
105	c.143T>C p.Leu48Ser	c.1241A>G p.Tyr414Cys	1089,7	350	cPKU	cPKU	60	LID	DBNS	LPD	LPD introduced from the first month, poorly controlled

106	c.473G>A p.Arg158Gln	c.898G>T p.Ala300Ser	320,8	N/A	MHP	MHP	100	AI	DBNS	ND	Not on LPD
107	c.1241A>G p.Tyr414Cys	c.441+5G>T	1271,3	320	cPKU	cPKU	95	AI	DBNS	LPD	LPD introduced from the first month, well controlled
108	c.1222C>T p.Arg408Trp	c.898G>T p.Ala300Ser	484,3	1000	MHP	MHP	100	AI	DBNS	LPD	LPD introduced from the first month, well controlled
109	c.143T>C p.Leu48Ser	c.782G>A p.Arg261Gln	1382	400	cPKU	cPKU	100	AI	DBNS	LPD	LPD introduced from the first month, well controlled
110	c.143T>C p.Leu48Ser	c.143T>C p.Leu48Ser	514,6	800	MHP	MHP	100	AI	DBNS	LPD	LPD introduced from the first month
111	c.143T>C p.Leu48Ser	c.898G>T p.Ala300Ser	363,22	N/A	MHP	MHP	100	AI	DBNS	ND	Not on LPD
112	c.473G>A p.Arg158Gln	c.898G>T p.Ala300Ser	440,29	600	MHP	MHP	90	AI	DBNS	LPD	LPD intrduced from the first month, poorly controlled
113	c.916A>G p.Ile306Val	c.916A>G p.Ile306Val	300	N/A	MHP	MHP	95	AI	DBNS	ND	Not on LPD
114	c.676C>A p.Gln226Lys	c.688G>A p.Val230Ile	294	N/A	MHP	MHP	105	HI	DBNS	ND	Not on LPD
115	c.143T>C p.Leu48Ser	c.143T>C p.Leu48Ser	786,97	450	mPKU	mPKU	120	HI	DBNS	LPD	LPD introduced from the first month
116	c.143T>C p.Leu48Ser	c.842C>T p.Pro281Leu	1029,12	350	mPKU	cPKU	70	BIF	DBNS	LPD	LPD introduced from the second month, well controlled, slightly delayed psychomotor development
117	c.143T>C p.Leu48Ser	c.1139C>T p.Thr380Met	258	N/A	MHP	MHP	105	HI	DBNS	ND	Not on LPD
118	c.916A>G p.Ile306Val	c.1222C>T p.Arg408Trp	544,83	380	MHP	mPKU	70	BIF	DBNS	LPD	LPD introduced from the first month, well controlled, delayed psychomotor development
119	c.473G>A p.Arg158Gln	c.1169A>G p.Glu390Gly	544,83	N/A	MHP	MHP	95	AI	DBNS	LPD	LPD introduced from the first month to 4 years of age, poorly controlled
120	c.842C>T p.Pro281Leu	c.1139C>T p.Thr380Met	300	N/A	MHP	MHP	95	AI	DBNS	ND	Not on LPD

121	c.143T>C p.Leu48Ser	c.143T>C p.Leu48Ser	≤ 850	350	mPKU	cPKU	70	BIF	DBNS	LPD	LPD introduced from the second month, delayed psychomotor development, poorly controlled
122	c.734T>C p.Val245Ala	c.916A>G p.Ile306Val	≤ 240	N/A	MHP	MHP	110	HI	DBNS	ND	Not on LPD
123	c.529G>C p.Val177Leu	c.441+5G>T	≤ 605,4	450	mPKU	mPKU	95	AI	DBNS	LPD	LPD introduced from the first month, well controlled
124	c.143T>C p.Leu48Ser	c.143T>C p.Leu48Ser	≤ 605,4	400	N/A	mPKU	N/A	N/A	DBNS	LPD	LPD well controlled, early psychomotor development is normal
125	c.1222C>T p.Arg408Trp	c.441+5G>T	2388	320	cPKU	cPKU	N/A	N/A	DBNS	LPD	LPD from the first month, well controlled, early psychomotor development is normal
126	c.473G>A p.Arg158Gln	c.898G>T p.Ala300Ser	≤ 320	N/A	MHP	MHP	95	AI	DBNS	ND	Not on LPD
127	c.1222C>T p.Arg408Trp	c.1208C>T p.Ala403Val	< 360	N/A	MHP	MHP	80	BIF	DBNS	ND	Not on LPD, premature birth, delayed psychomotor development
128	c.143T>C p.Leu48Ser	c.143T>C p.Leu48Ser	>1200	>600	cPKU	MHP	84	BIF	LD	ND	Not on LPD
129	c.143T>C p.Leu48Ser	c.143T>C p.Leu48Ser	>1200	>600	cPKU	MHP	82.5	BIF	LD	ND	Not on LPD
130	c.1222C>T p.Arg408Trp	c.1247C>A p.Pro416Gln	1041,2	N/A	mPKU	mPKU	N/A	LID	LD	ND	Diagnosed after her child was diagnosed with PKU
131	c.782G>A p.Arg261Gln	c.916A>G p.Ile306Val	246	N/A	MHP	MHP	N/A	N/A	LD	ND	N/A

Maximal Phe level (Max Phe) represents the highest Phe pretreatment blood concentration (μmol/L); The three PKU phenotypes are: cPKU – classic PKU; mPKU – moderate PKU; MHP – mild hyperphenylalaninemia. Abbreviations used in the table: DBNS – diagnosis by neonatal screening; LD – late diagnosis; LPD – low Phe diet; PCD – poorly controlled diet; ND – no diet; NND – no need for diet; SID – Severe intellectual disability (IQ 20-40); MID – Moderate intellectual disability (IQ 40-55); LID – Mild intellectual disability (IQ 55-68); BIF – Borderline intellectual functioning (IQ 68-84); LAI – Low average intelligence (IQ 84-90); AI – Average intelligence (IQ 90-100); HI – High intelligence (IQ >100).

Table S2. BH4-responsiveness based on genotype for Serbian PKU patients

		Genotype	Number of patients with each genotype
**BH4 responsive**	R + R	p.Val245Ala; p.Ile306Val	2
		p.Ile306Val; p.Ile306Val	1
		p.Ile306Val; p.Ala403Val	1
		p.Glu390Gly; p.Glu390Gly	1
	R + IR	p.Leu48Ser; p.Ile306Val	3
		p.Arg158Gln; p.Ala300Ser	3
		p.Arg261Gln; p.Ile306Val	3
		p.Leu48Ser; p.Val245Ala	2
		p.Leu48Ser; p.Ala300Ser	2
		p.Arg158Gln; p.Glu390Gly	2
		p.Leu48Ser; p.Tyr414Cys	1
		p.Arg261Gln; p.Glu390Gly	1
	R + N	p.Glu390Gly; p.Arg408Trp	4
		p.Val177Leu; p.Ala403Val	3
		p.Ile306Val; p.Arg408Trp	3
		p.Ala403Val; p.Arg408Trp	2
		c.441+5G>T; p.Ile306Val	1
		c.441+5G>T; p.Tyr414Cys	1
		c.442-5C>G; p.Tyr414Cys	1
		c.(441+1_442-1)_(509+1_510-1)del; p.Ile306Val	1
		c.1066-11G>A; p.Ala403Val	1
		c.1066-11G>A; p.Tyr414Cys	1
		p.Leu213Pro; p.Tyr414Cys	1
		p.Pro225Thr; p.Ile306Val	1
		p.Gln226Lys; p.Val230Ile	1
		p.Gln226Lys; p.Ile306Val	1
		p.Arg261Ter; p.Ile306Val	1
		p.Arg261Ter; p.Glu390Gly	1
		p.Tyr268Cys; p.Ile306Val	1
		p.Pro281Leu; p.Glu390Gly	1
		p.Ala300Ser; p.Arg408Trp	1
		p.Ile306Val; p.Pro416Gln	1

Probably BH4 responsive	IR + IR	p.Leu48Ser; p.Leu48Ser	20
		p.Leu48Ser; p.Arg158Gln	3
		p.Leu48Ser; p.Arg261Gln	3
		p.Leu48Ser; p.Thr380Met	1
	IR + N	p.Leu48Ser; p.Arg408Trp	9
		p.Leu48Ser; p.Val177Leu	4
		p.Leu48Ser; p.Pro281Leu	4
		c.442-5C>G; p.Leu48Ser	2
		p.Leu48Ser; p.Ser231Phe	2
		c.1315+1G>A; p.Arg261Gln	1
		p.Ser16Ter; p.Arg261Gln	1
		p.Gln20Ter; p.Leu48Ser	1
		p.Leu48Ser; p.Pro225Thr	1
		p.Leu48Ser; p.Arg261Ter	1
		p.Leu48Ser; p.Pro416Gln	1
		p.Arg261Gln; p.Arg408Trp	1
		p.Pro281Leu; p.Thr380Met	1
		p.Arg408Trp; p.Arg413Pro	1
Non-BH4 responsive	N + N	p.Arg408Trp; p.Arg408Trp	2
		p.Pro225Thr; p.Pro281Leu	1
		c.441+5G>T; p.Pro281Leu	1
		c.441+5G>T; p.Val177Leu	1
		c.441+5G>T; p.Arg408Trp	1
		c.(509+1_510-1)_(706+1_707-1)del; c.(509+1_510-1)_(706+1_707-1)del	1
		c.1065+3A>G; p.Arg408Trp	1
		c.1066-11G>A; p.Arg111Ter	1
		c.1315+1G>A; p.Pro281Leu	1
		c.1315+1G>A; p.Arg297His	1
		p.Gln20Ter; p.Val177Leu	1
		p.Gln20Ter; p.Gln226Lys	1
		p.Arg111Ter; p.Arg111Ter	1
		p.Val177Leu; p.Arg408Trp	1
		p.Pro225Thr; p.Arg408Trp	1
		p.Arg243Ter; p.Arg243Ter	1
		p.Arg252Gln; p.Arg408Trp	1
		p.Pro281Leu; p.Pro281Leu	1
		p.Lys363AsnfsTer37; p.Arg408Trp	1
		p.Arg408Trp; p.Pro416Gln	1

PAH variants are classified as: R – responsive, IR – inconsistently responsive, and N – nonresponsive (or unknown to be responsive). Genotypes with one unidentified variant were excluded from the table.

Table S3. Primers used for *PAH* gene amplification and sequencing.

Primer name	Primer sequence (5’-3’)	Amplified region
PAH1F	TTAAAACCTTCAGCCCCACG	Exon 1
PAH1R	TGGAGGCCCAAATTCCCCTAACTG	
PAH2F	GAGGTTTAACAGGAATGAATTGCT	Exon 2
PAH2R	TCCTGTGTTCTTTTCATTGC	
PAH3F	GCCTGCGTTAGTTCCTGTGA	Exon 3
PAH3R	CTTATGTTGCAAAATTCCT	
PAH4F	ATGTTCTGCCAATCTGTACTCAGGA	Exon 4
PAH4R	CAAGACACAGGCCATGGACT	
PAH5F	TCATGGCTTTAGAGCCCCCA	Exon 5
PAH5R	AGGCTAGGGGTGTGTTTTTC	
PAH6F	CCGACTCCCTCTGCTAACC	Exon 6
PAH6R	CAATCCTCCCCCAACTTTCT	
PAH7F	GGTGATGAGCTTTTAGTTTTCTTTC	Exon 7
PAH7R	AGCAATGAACCCAAACCTC	
PAH8F	TGGCTTAAACCTCCTCCCCT	Exon 8
PAH8R	CTGGGCTCAACTCATTTGAG	
PAH9F	CCGATGTCACCACTTATCTGC	Exon 9
PAH9R	GTTGGTGGGTTCAAGATACTGC	
PAH10F	TTAACCATCATAGAGTGTGC	Exon 10
PAH10R	ACAAATAGGGTTTCAACAAT	
PAH11F	TGAGAGAAGGGGCACAAATG	Exon 11
PAH11R	GCCAACCACCCACAGATGA	
PAH12F	ATGCCACTGAGAACTCTCTT	Exon 12
PAH12R	ACTGAGAAACCGAGTGGCCT	
PAH13F	GACACTTGAAGAGTTTTTGC	Exon 13
PAH13R	TTTTCGGACTTTTTCTGATG	
PAH prom FW	CAGCAAGGCAGTGTGCTTAG	Promoter
PAH prom RV	CTCAGGTACAGGCAGGTTTG	
